# Synthesis and Neuroprotective Action of Optically Pure Neoechinulin A and Its Analogs

**DOI:** 10.3390/ph3041063

**Published:** 2010-03-31

**Authors:** Toshiaki Aoki, Kensuke Ohnishi, Masaaki Kimoto, Satoshi Fujieda, Kouji Kuramochi, Toshifumi Takeuchi, Atsuo Nakazaki, Nobuo Watanabe, Fumio Sugawara, Takao Arai, Susumu Kobayashi

**Affiliations:** 1 Faculty of Pharmaceutical Sciences, Tokyo University of Science, 2641 Yamazaki, Noda, Chiba, 278-8510, Japan; 2 Genome and Drug Discovery Center, Tokyo University of Science, 2641 Yamazaki, Noda, Chiba, 278-8510, Japan; 3 Department of Applied Biological Science, Tokyo University of Science, 2641 Yamazaki, Noda, Chiba, 278-8510, Japan

**Keywords:** neoechinulin A, intramolecular cyclization, cytoprotective activity

## Abstract

We developed an efficient, stereoselective synthetic method for the diketopiperazine moiety of neoechinulin A and its derivatives. The intramolecular cyclization at 80 ºC proceeded with minimal racemization of the stereogenic center at C-12 on neoechinulin A, even though the cyclization at 110 ºC caused partial racemization. In contrast with these results, the cyclization on diketopiperazine of 8,9-dihydroneoechinulin A derivatives did not cause epimerization of the stereogenic centers, even at 110 °C. We examined the structure-activity relationships for the cytoprotective activity against cytotoxicity induced by 3-morpholinosydnonimine (SIN-1) in nerve growth factor (NGF)-differentiated PC12 cells. The C-8/C-9 double bond, but not the stereogenic center derived from alanine, was found to play a key role in the cytoprotective activity.

## 1. Introduction

**Figure 1:**
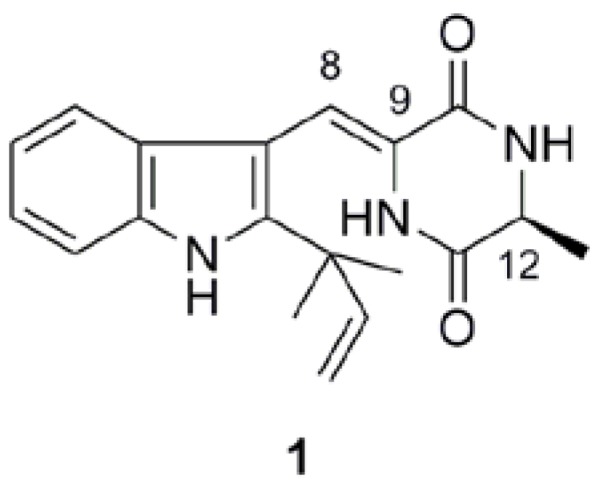
The structure of natural neoechinulin A (**1**).

Diketopiperazines have various therapeutically important biological properties such as antitumor, antiviral, antifungal, and antihyperglycaemic activities [1]. Neoechinulin A (Figure 1), an indole alkaloid containing diketopiperazine, has been isolated from marine fungi including *Aspergillus sp.*, and is also reported to have antitumor activity [2]. We found that neoechinulin A (**1**) displays cytoprotective activity in neuron-like PC12 cells against oxidative insults induced by peroxynitrite generated from 3-(4-morpholinyl)sydnonimine hydrochloride (SIN-1) [3]. Subsequently we examined the structure-activity relationship of neoechinulin A analogs with anti-nitration activity, anti-oxidant activity and cytoprotective activity against peroxynitrite from SIN-1 in PC12 cells [4,5]. As a result of these studies the structural characteristics required for cytoprotection were determined. Recently, we found that **1** could also protect PC12 cells from cytotoxicity of 1-methyl-4-phenylpyridinium (MPP^+^), a neurotoxin capable of provoking acute Parkinson-like neurodegeneration in humans. This observation raises the possibility that neoechinulin A or its analogs may have therapeutic utility for the treatment of neurodegenerative disorders [6].

We have previously achieved the total synthesis of **1** with high enantiomeric excess and determined its absolute configuration [7,8]. The key step in the synthesis is the intramolecular cyclization of the ΔTrp-L-Ala derivative (**2**) to construct the diketopiperazine ring. However, during the synthesis of optically pure neoechinulin A, the stereogenic center on the diketopiperazine moiety was subject to epimerization. In this paper, we report efficient, stereoselective synthetic methods for the diketopiperazine moiety of neoechinulin A and its derivatives.

## 2. Results and Discussion

### 2.1. Preparation of neoechinulin A and its analogs *via* intramolecular cyclization

**Scheme 1:**
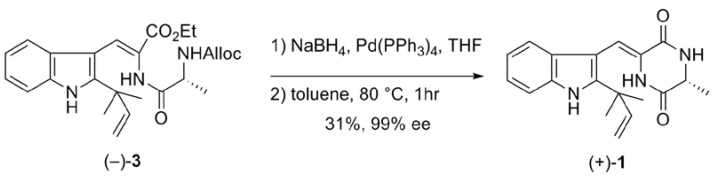
Preparation of optically pure (+)-neoechinulin A.

We prepared optically active neoechinulin A from the cyclization precursor **2**. The Alloc group of (+)**-3** was removed by treatment with NaBH_4_ and a catalytic amount of Pd(PPh_3_)_4_ in THF to afford amine **2** [7,8]. We then investigated the thermally induced formation of diketopiperazine (Table 1). When a solution of amine **2** in toluene was heated at 110 ºC for 1 hr, (–)-**1** was obtained from **3** in 43% yield with 81% ee (entry 1). The cyclization was also conducted at 80 ºC to afford (–)-**1** in 58% yield from **3** with 95% ee (entry 2) [7,8]. Thus, the stereogenic center of (–)-**1** was partially epimerized at the elevated temperature. According to the same procedure as entry 2 in Table 1, we synthesized (+)-**1**, the enantiomer of natural neoechinulin A, with 99% ee from ΔTrp-D-Ala derivative ((–)**-3**) (Scheme 1).

**Table 1:** Preparation of optically pure (–)-neoechinulin A. 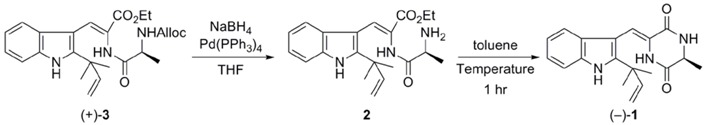

**Entry**	**Temperature (ºC)**	**Yield (%)** *^a^*	**ee (%)** *^b^*
1	110	43	81
2*^c^*	80	58	95

*^a^* Isolated yield of (–)-**1** from (+)-**3**. *^b^* The enantiomeric excess was determined by chiral HPLC (DAICEL AD-H; hexane : 2-propanol = 9 : 1).*^c^* Adapted from [7,8]

**Table 2:** Preparation of preechinulin (**4**). 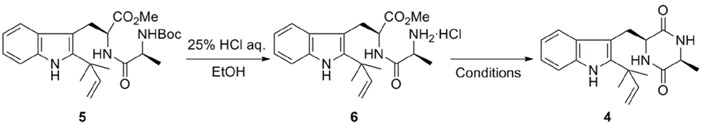

**Entry**	**Solvent**	**Temperature (ºC)**	**Time (days)**	**Yield (%)*^a^***
1*^b^*	benzene	80	7	73
2	toluene	110	1	83

*^a^* Isolated yield of **4** from **5**. *^b^* Adapted from [5].

**Scheme 2:**
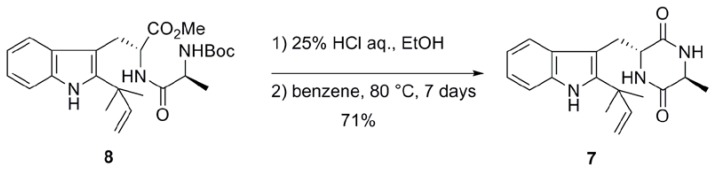
Preparation of compound **7**(Adapted from [5]).

We prepared preechinulin (**4**) from **5** according to a similar procedure (Table 2). Removal of the *N*-Boc protective group in **5** by aqueous HCl solution in EtOH followed by thermal cyclization of **6** in benzene at 80 ºC for 7 days gave **4** as a single isomer in 73% yield (entry 1) [5]. Because the diastereomers of **4** were not observed, it seems that no epimerization took place during the reaction. When the cyclization was performed in toluene at 110 ºC for 1 day, compound **4 **was obtained in 83% yield as a sole product (entry 2). We also prepared **7**, a diastereomer of preechinulin, from D-Trp-L-Ala derivative **8 **by heating at 80 ºC (Scheme 2) [5]. In this case, **7** was also obtained as a single isomer.

We found that the stereogenic center on the diketopiperazine moiety of **1** was partially racemized at high temperature. The partial racemization of **1** proceeded much faster than that of 8,9-dihydroneoechinulin A derivatives (**4 **and **7**). These results suggest that the acidity of the α-proton of the alanine moiety in **1** might be greater than that in **4 **or **7**. 

### 2.2. Biological and chemical properties of optical isomers of neoechinulin A and preechinulin

Our previous studies demonstrated that neoechinulin A treatment affords cytoprotection against oxidative/nitrosative insults imposed by the O_2_^-^/NO donor SIN-1 to neuronal (-like) cells, such as nerve growth factor (NGF)-differentiated PC12 cells and rat primary brain neurons, but not to non-neuronal cells, such as undifferentiated PC12 cells and fibroblasts [2]. In addition, the cytoprotection requires at least a 12-hr pretreatment of cells with neoechinulin A before challenging with SIN-1, suggesting that the cytoprotection conferred by neoechinulin A probably depends on induction of certain cytoprotective genes [3,4]. Here we summarize that cytoprotective activity of those compounds against cytotoxicity induced by SIN-1 in NGF-differentiated PC12 cells (Table 3). Treatment of PC12 cells with (–)-**1** or (+)-**1** for 24 hr gave almost identical levels of protection against cytotoxicity when subsequently challenged with SIN-1 [4]. In contrast, neither **4** nor **7** showed any cytoprotection against SIN-1 [4]. These results suggest that the orientation of the methyl group at C-12 or the isoprenylated indole group at C-9 does not contribute to cytoprotection; rather the presence of the C-8/C-9 double bond is essential for the cytoprotective action of neoechinulin A [4,5].

Additionally, both (–)-**1** and (+)-**1** also potently inhibited nitrotyrosine formation in BSA induced by the addition of SIN-1 to a cell-free system. By contrast, neither **4** or **7** displayed any inhibition on nitrotyrosine formation under otherwise identical conditions [4,5]. Although inhibition of nitrotyrosine formation is not essential for cytoprotection against SIN-1, the C-8/C-9 double bond could play a pivotal role in inhibition of both SIN-1-induced tyrosine nitration and SIN-1-induced PC12 cell death irrespective of the orientation of the methyl group at C-12 [4].

**Table 3:** Cytoprotective activity of neoechinulin A stereoisomers against SIN-1-induced cell death in NGF-differentiated PC12 cells. ^a^

**Compound**	**Viability (%)**	**Relative protection (% vs. (+)-1)**	**Reference**
vehicle	31.4 ± 2.0	0	[4]
(–)**-1**	89.6 ± 11.2*^b^*	113.9	
(+)**-1**	82.5 ± 11.6*^b^*	100.0	
vehicle	9.7 ± 13.2	0	[5]
(–)**-1**	49.7 ± 8.3*^b^*	100	
**4**	7.0 ± 7.0	–6.8	
**7**	12.5 ± 10.2	7.1	

*^a ^*PC12 cells were differentiated with NGF for 3 days, followed by treatment with vehicle (control) or each compound for an additional 24 hr, and then challenged with SIN-1 for 24 hr. Viability (% of non-SIN-1-treated cells) was assessed by either cell-counting kit-8 or LDH assay. The values were expressed as mean ± S.D. from at least three independent experiments. [4,5]. *^b^* p < 0.01 *versus* respective vehicle-treated control.

## 3. Experimental Section

### General

All reactions were monitored by TLC, which was carried out on Silica Gel 60 F254 plates (Merck). Flash chromatography separations were performed on PSQ 100B (Fuji Silysia Co., Ltd., Japan). The NMR spectra (^1^H and ^13^C) were recorded on a Bruker 600 MHz or 400 MHz spectrometer (Avance DRX-600, Avance DRX-400) or a JEOL 400 MHz spectrometer (JNM-LD400), using CDCl_3_ solutions (with TMS for ^1^H-NMR and chloroform-*d* for ^13^C-NMR as the internal reference), unless otherwise noted. Optical rotations were recorded on a JASCO P-1030 digital polarimeter at room temperature, using the sodium D line.

*(–)-Neoechinulin A* ((–)-**1**)* obtained by cyclization of* **2*** at 110 ºC in toluene (*Table 1*, Entry 1).* To a solution of (+)-**3** (8.2 mg, 18 μmol) in dry THF (0.5 mL) was added NaBH_4_ (2.7 mg, 72 μmol) and Pd(PPh_3_)_4_ (0.42 mg, 0.36 μmol) at 0 °C. The reaction mixture was stirred at room temperature for 1 hr. The reaction was quenched with saturated aqueous NH_4_Cl, filtered through Celite. The aqueous layer was extracted with CHCl_3_, and the combined extracts were washed with brine, dried over Na_2_SO_4_, and concentrated *in vacuo* to afford crude **2** (7.9 mg). A solution of **2** (7.9 mg) in toluene (0.5 mL) was stirred at 110 °C for 1 hr and the reaction mixture was then concentrated *in vacuo*. The residue was purified by flash chromatography (hexane-EtOAc = 1:3) to afford (−)-**1** (2.5 mg, 43% from (+)-**3**) as needle-like crystals. The ^1^H- and ^13^C-NMR spectra of (–)-**1** were identical with those of (–)-**1** [7,8]. Enantiomeric excess of (–)-**1** was 81% analyzed by HPLC using chiral column (DAICEL AD-H) eluted with a mixture of hexane and 2-propanol (9:1). 

*(+)-Neoechinulin A* ((+)**-1**). To a solution of (–)-**3** (50.2 mg, 0.11 mmol) in dry THF (1.1 mL) was added NaBH_4_ (16.8 mg, 0.44 mmol) and Pd(PPh_3_)_4_ (2.6 mg, 2.2 μmol) at 0 °C. The reaction mixture was stirred at room temperature for 1 hr. The reaction was quenched with saturated aqueous NH_4_Cl, filtered through Celite. The aqueous layer was extracted with CHCl_3_, and the combined extracts were washed with brine, dried over Na_2_SO_4_, and concentrated *in vacuo* to afford crude amine (43.6 mg). A solution of the amine (43.6 mg) in toluene (2.5 mL) was heated at 80 °C for 1 hr and the reaction mixture was then concentrated in vacuo. The residue was purified by flash chromatography (hexane:EtOAc = 1:3) to afford (+)-**1** (11.0 mg, 31% from (+)-**3**) as needle-like crystals. The ^1^H- and ^13^C-NMR spectra of (+)-**1** were identical with those of (–)-**1** [7,8]. Enantiomeric excess of (+)-**1** was 99% analyzed by HPLC using chiral column (DAICEL AD-H) eluted with a mixture of hexane and 2-propanol (9:1). [α]_D_^22^ = +56 (c 0.1, MeOH). 

*Preechinulin* (**4**)* obtained by cyclization of* **6*** at 110 ºC in toluene (*Table 2*, Entry 2).* To a solution of **5** (8.1 mg, 0.017 mmol) in EtOH (2.2 mL) was added a 25% aqueous solution of HCl (1.1 mL), and the mixture was stirred at rt for 2.5 hr. The mixture was concentrated to give a crude ammonium salt. A solution of the crude ammonium salt in toluene (1.8 mL) was stirred at 110 °C for 1 day. The mixture was concentrated to give a crude residue, which was purified by a preparative TLC (hexanes-toluene- isopropylamine = 5:3:2) to afford **5** (4.8 mg, 83%) as a white solid. The ^1^H- and ^13^C-NMR spectra of **4** were identical with those reported [5].

## 4. Conclusions

We prepared (–) and (+)-neoechinulin A (**1**) with high enantiomeric excess by the intramolecular cyclization of **2** and its enantiomer, respectively at 80 °C. The thermal cyclization at 110 °C caused partial epimerization of the stereogenic center at C-12 in **1**. By contrast, 8,9-dihydroneoechinulin A derivatives **4** and **7** were obtained in optically pure forms by a similar cyclization at 110 °C. These results suggest that the acidity of the α-proton of the L-Ala moiety in **1** might be greater than that in either **4 **or **7**. We found that both (–) and (+)-**1** protected the cells against cytotoxicity to an almost identical extent following exposure to SIN-1. In contrast, neither **4** nor **7** showed any cytoprotection against SIN-1. These results indicate that the C-8/C-9 double bond could play a pivotal role in inhibition of both SIN-1-induced tyrosine nitration and SIN-1-induced PC12 cell death irrespective of the orientation of the methyl group at C-12.
